# A virus that has gone viral: amino acid mutation in S protein of Indian isolate of Coronavirus COVID-19 might impact receptor binding, and thus, infectivity

**DOI:** 10.1042/BSR20201312

**Published:** 2020-05-15

**Authors:** Priyanka Saha, Arup Kumar Banerjee, Prem Prakash Tripathi, Amit Kumar Srivastava, Upasana Ray

**Affiliations:** 1 Cancer Biology and Inflammatory Disorder Division, CSIR-Indian Institute of Chemical Biology, 4, Raja S.C., Mullick Road, Jadavpur, Kolkata 700032, West Bengal, India; 2 Department of Biochemistry, North Bengal Medical College and Hospital, Sushrutanagar, Siliguri 734012, West Bengal, India; 3 Cell Biology and Physiology Division, CSIR-Indian Institute of Chemical Biology, 4, Raja S.C., Mullick Road, Jadavpur, Kolkata 700032, West Bengal, India; 4 Infectious Biology and Immunology Division, CSIR-Indian Institute of Chemical Biology, 4, Raja S.C., Mullick Road, Jadavpur, Kolkata 700032, West Bengal, India

**Keywords:** Coronavirus, COVID-19, RBD, S protein, SARS-CoV-, virology

## Abstract

Since 2002, β coronaviruses (CoVs) have caused three zoonotic outbreaks, SARS-CoV in 2002, MERS-CoV in 2012, and the recent outbreak of SARS-CoV-2 late in 2019 (also named as COVID-19 or novel coronavirus 2019 or nCoV2019). Spike (S) protein, one of the structural proteins of this virus plays key role in receptor (ACE2) binding and thus virus entry. Thus, this protein has attracted scientists for detailed study and therapeutic targeting. As the nCoV2019 takes its course throughout the world, more and more sequence analyses are being done and genome sequences are being deposited in various databases. From India, two clinical isolates have been sequenced and the full genome has been deposited in GenBank. We have performed sequence analyses of the Spike protein of the Indian isolates and compared with that of the Wuhan, China (where the outbreak was first reported). While all the sequences of Wuhan isolates are identical, we found point mutations in the Indian isolates. Out of the two isolates, one was found to harbor a mutation in its receptor-binding domain (RBD) at position 407. At this site, arginine (a positively charged amino acid) was replaced by isoleucine (a hydrophobic amino acid that is also a C-β branched amino acid). This mutation has been seen to change the secondary structure of the protein at that region and this can potentially alter receptor binding of the virus. Although this finding needs further validation and more sequencing, the information might be useful in rational drug designing and vaccine engineering.

## Introduction

A virus gone viral. First case of COVID-19 was reported in December 2019 in Wuhan (China) and since then it has spread worldwide becoming a pandemic, with maximum death cases in Italy, although initially, the maximum mortality was reported from China [1]. According to a World Health Organization (WHO) report, as of 2 April 2020, there were confirmed 823626 COVID-19 cases and 40598 deaths, that included cases which were both locally transmitted or imported [2]. There are published reports which suggest that SARS-CoV-2 shares highest similarity with bat SARS-CoV [3]. Scientists across the globe are trying to elucidate the genome characteristics using phylogenetic, structural, and mutational studies [4]. Spike (S) protein, one of the key proteins of SARS-CoV-2 is involved directly with virus infection as it is involved in receptor recognition, attachment, binding, and entry [5–7]. S protein has two major domains, S1 and S2 [6]. S1 helps in attachment and binding to the host cell receptor, while S2 mediates fusion to the host cell membrane. Thus, both these domains play crucial roles in establishing successful entry of the virion into its host cell. Coronaviruses (CoVs) are known to mutate rapidly especially, the Spike protein. Mutations help the virus to escape host cell immune surveillance thereby acclimatizing with the host environment. Mutations in the Spike protein might also lead to emergence of mutants or variants which have elaborate cellular tropism or altered virulence. Ultimately mutations help the virus to evolve into a better version of itself that fit best in its host environment. Since, Spike protein is one of the major targets for drug and vaccine designing, sequence analyses of the Spike protein can give us a plethora of information which can be instrumental in rational drug and vaccine development. In the present piece of work, we retrieved S protein sequences of the SARS-CoV-2 from different geographical locations to identify notable features of S protein especially in Indian isolates. These analyses include identification of mutational signatures and their correlation with virus infection. Our analyses show unique point mutations in the Spike protein of the Indian subtypes.

## Methods

### Sequence source

Since COVID 19 or SARS-CoV-2 started from Wuhan, China, we started our analyses with Spike protein sequences from Wuhan. For our study, we considered all the full-length sequences that were available in GenBank. We first compared 17 available S protein sequences from Wuhan. Since they showed 100% sequence similarities, we considered one of these for our further analyses. Since Italy has also been affected aggressively by COVID-19, we included the sequence in our study. In the present paper we have focussed on the first two deposited COVID-19 isolates from India (MT012098 and MT050493).

### Sequence analyses

For our sequence alignments, we have used NCBI BLAST, CLUSTAL W, and CLUSTAL OMEGA. To predict secondary structure, we have used CFSSP (Chou and Fasman secondary structure prediction) server.

MutPred server was used to analyze the mutation. JMol and ConSurf tools were used to predict the structure of the proteins. PyMoL standalone software was used to visualize the structure and understand the pattern of bonding. Further kinetics and structure analyses were performed by the DynaMut Server and Chimera version 11.

## Results and discussion

SARS-CoV-2 sequence data are expanding rapidly in the databases as the virus spreads worldwide. Although many sequences from various countries have been deposited, limited full genome sequences are available from most of the countries. This virus has infected people in various countries like China, Italy, Spain, U.S.A., Germany, France, United Kingdom, India and many more and the data are updated almost regularly by the WHO. As of now, compared with many countries, the rate of transmission is comparatively controlled in India. Although this might be influenced by many factors like general immunity, point of entry of this virus in the country, measures taken to contain the spread, diagnosis, data management, etc., we have used the available sequence data of Indian isolates to understand the biology of this virus.

**Figure 1:**
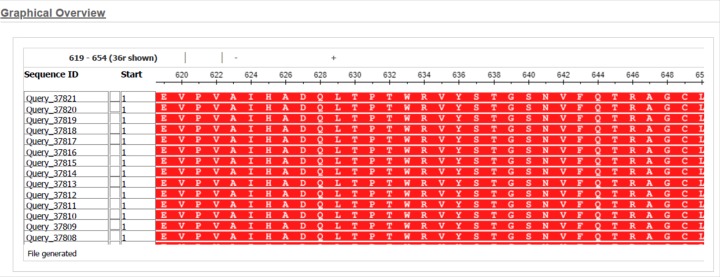
Multiple sequence alignment of Spike protein sequences of Wuhan isolates Seventeen Spike protein sequences available in GenBank were aligned using NCBI BLASTp online tool and the multiple sequence alignment result has been shown.

From India, there were only two full genome sequences submitted from the state of Kerala (GenBank accession numbers MT012098 {(isolate SARS-CoV-2/human/IND/29/2020 or isolate 29) and MT050493 (isolate SARS-CoV-2/human/IND/166/2020 or isolate 166)}. We have compared the S protein sequence from these two isolates with that of Wuhan. All the 17 sequences from Wuhan that were first aligned to check sequence variability showed 100% sequence similarities (Figure 1).

**Figure 2:**
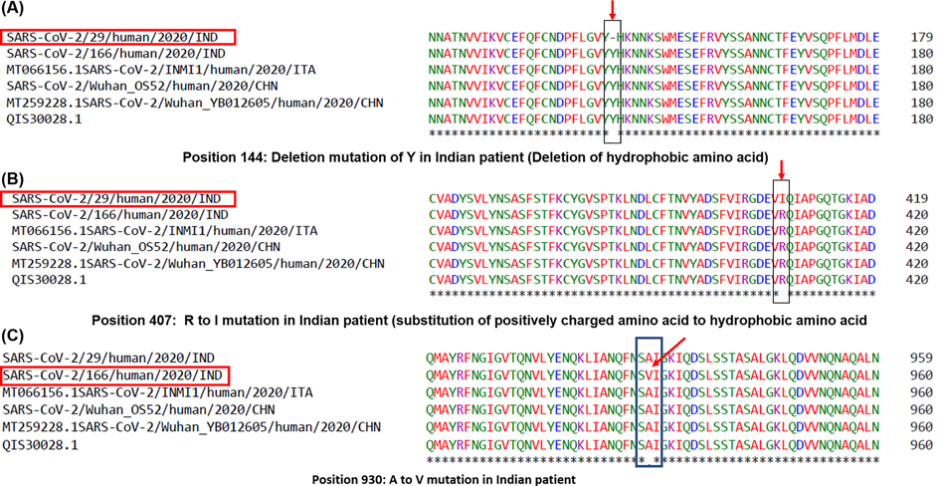
Multiple sequence alignment of Spike protein sequences of Indian, Wuhan, and Italian isolates Spike protein sequences of Indian isolates available in GenBank were aligned using CLUSTAL Omega online tool with color coding option and the multiple sequence alignment result has been shown above. The Indian isolates having mutations have been highlighted with red boxes. The mutations have been marked with red arrows. (**A**) Deletion mutation of Y in isolate 29. (**B**) Substitution mutation R→I at position 407 in isolate 29. (**C**) Substitution mutation of A→V at position 930 in isolate 166.

To compare the Indian isolates, we aligned the S protein sequences of these isolates with Wuhan isolates and a sequence from Italy. While Wuhan and Italian isolates matched completely, we found few mutations in case of Indian isolates 29 and 166 as shown in Figure 2.

**Figure 3:**
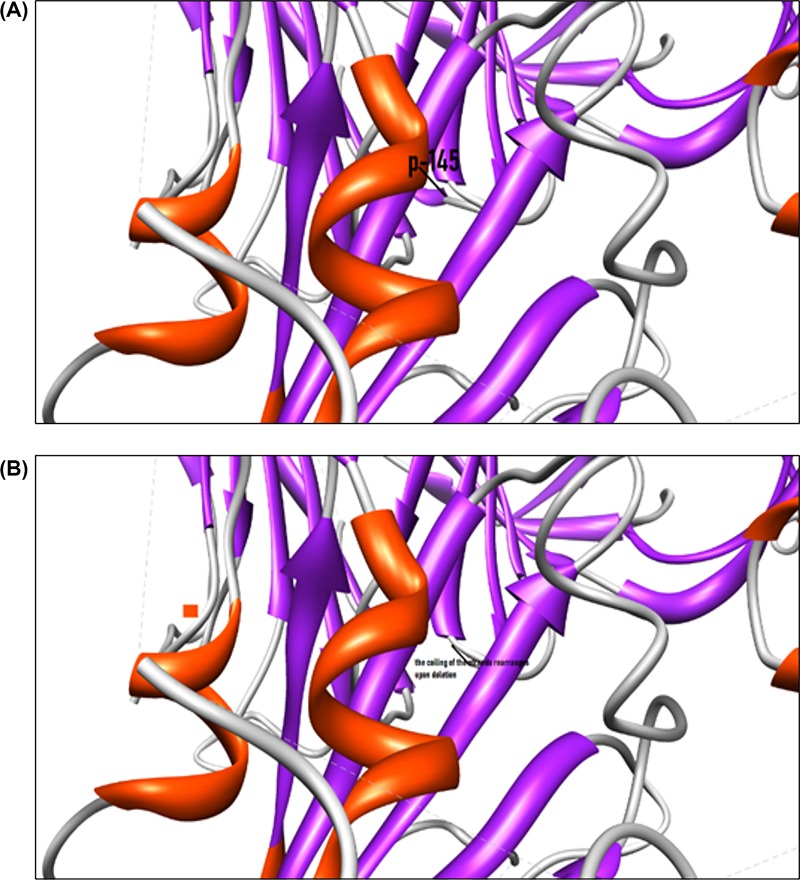
Deletion of the amino acid in 144 in Indian isolate 29 (**A**) Shows structure without deletion of amino acid tyrosine at position 144. (**B**) Shows coiling of the strands rearranged after the deletion.

We observed that isolate 29 had two mutations, a deletion mutation where Y (tyrosine) at position 144 was absent as compared with the Wuhan and Italian isolates. Due to deletion of the amino acid residues in position 144 in the protein structure, there is a change in the β sheets (Figure 3). This alteration may change the orientation of the molecule and also the stability of the protein itself. Ramachandran plot of this structure shows slight shifts of angle in the β-sheet configuration.

On the other hand, at position 407, the same isolate had a substitution mutation of R (Arginine) to I (Isoleucine) (R407I). The receptor-binding domain or RBD of the Spike protein of SARS-CoV-2 lie between amino acids 331 and 524 [8]. Thus, the mutation R407I lies in the RBD, which plays key role in receptor binding. Arginine is a positively charged amino acid and Isoleucine is a hydrophobic amino acid with C-β branch. While positively charged amino acid could be more exposed, hydrophobic amino acids secure themselves away from the outer aqueous environment. Since nature of these amino acids are so different, a substitution of this nature might change the conformation locally and can impose functional alterations, i.e. with respect to receptor interaction. To confirm this theory further, we ran a secondary structure prediction using CFSSP server and found that while in case of Wuhan isolate, this region had helix only (H) (Figure 4A), in case of R407I in Indian isolate there was introduction of sheets (E) (Figure 4B). This suggests that a change in secondary structure occurs in case of RBD of spike protein of isolate 29 of the 2019 novel Coronavirus (nCoV2019) of India. Tertiary structure analyses showed that there because of the mutation there is an introduction of additional oxygen molecule to the next residue. The protein stability score drops sharply (−4.08) and thereby its electrostatic force. Such a condition makes the protein flexible and might affect interaction with the receptor. Alteration to the structure will cause shift in the hydrogen bonds and also the bond angle, two main prerequisites for strong interaction with the receptor. The hydrogen potential tends to increase from 10 to 13.2 in case of mutation.

**Figure 4A:**
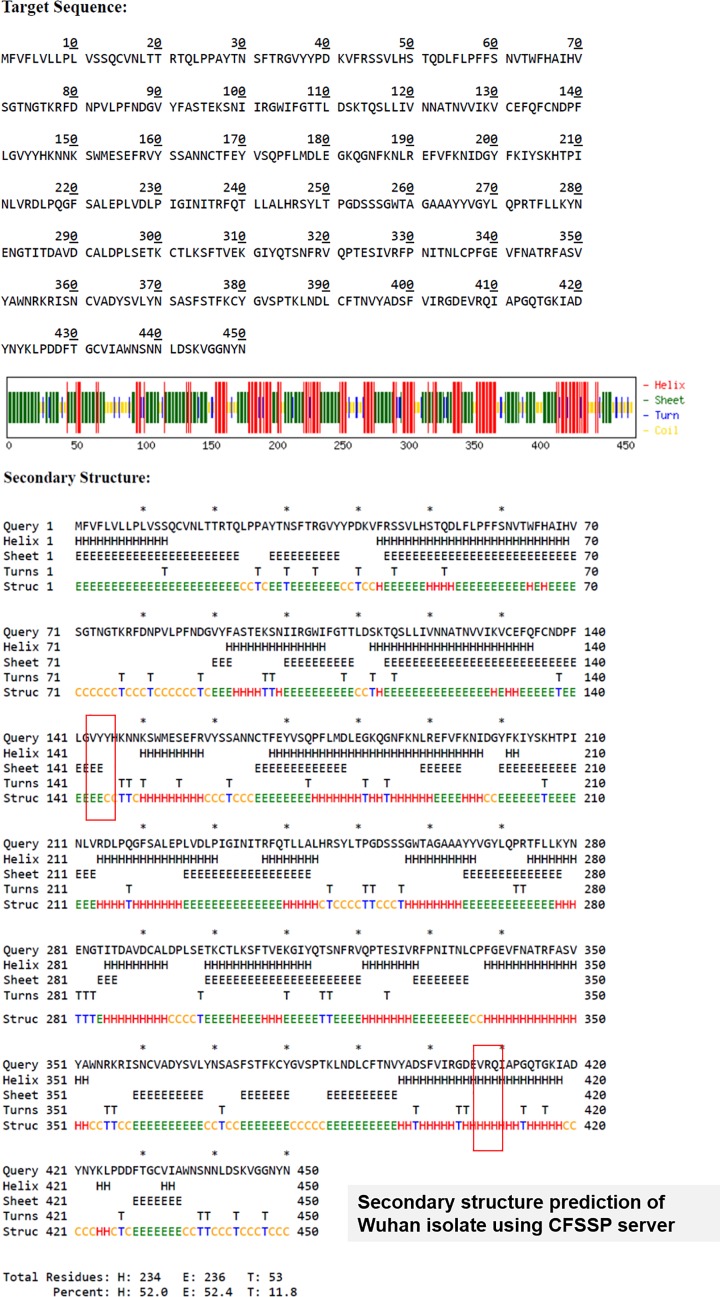
Secondary structure prediction of spike protein of Wuhan isolate The area marked in red box has helix (H). This area has been seen to get mutated in Indian isolate

**Figure 4B:**
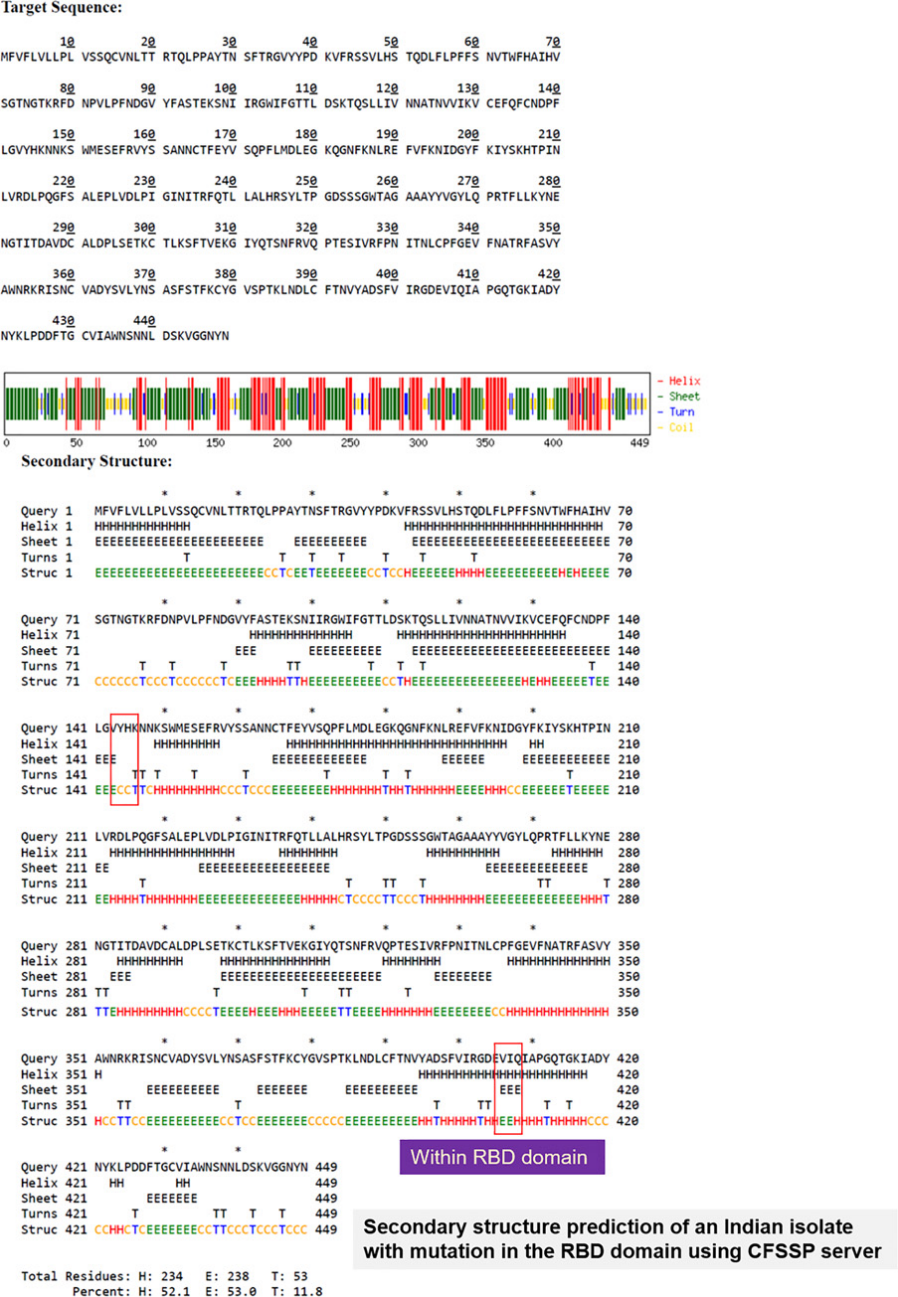
Secondary structure prediction of spike protein of Indian isolate 29 The area marked in red box that had helix (H) in Wuhan isolate shows introduction of sheets (E).

**Figure 5:**
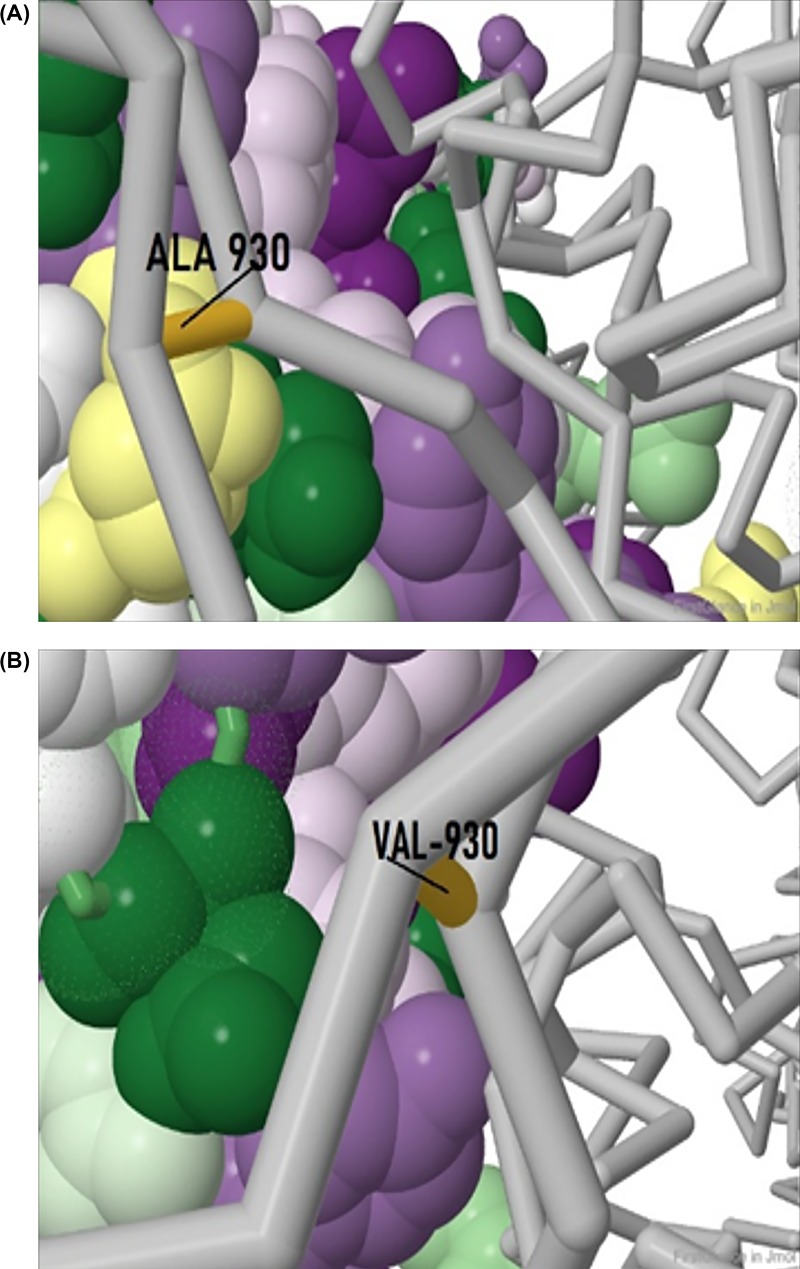
Spike protein mutation in isolate 166 Alanine (**A**) to Valine (**B**) mutation in isolate 166.

For the isolate 166 of India, we found a different mutation at position 930 of the spike protein (Figure 2C). Herein, there was a substitution of A (alanine) to V (valine) at position 930 (A930V). Since both the amino acids are hydrophobic in nature, any change that might occur due to this mutation might get masked upon tertiary structure formation and thus not impose a functional change in the protein, i.e. a conservative mutation. Despite this possibility, valine has some unique characteristics. Valine is one of the C-β branched amino acids like threonine and isoleucine. C-β branched amino acids are bulkier toward the main chain and it is difficult for them to attain α-helical conformations. Such amino acids have restricted conformations, are destabilizing in nature causing distortion in local helix backbone [9]. S protein if SARS-CoV2 has two domains: S1 and S2 [8]. While S1 has the RBD and in involved in receptor binding, S2 mediates fusion of viral and host cell membranes. Mutation A930V of Indian isolate 166 falls in the S2 subunit of S protein. Considering the nature of valine being destabilizing causing distortion, this mutation might have implications in viral membrane fusion subject to validation. Structure of the spike glycoprotein was retrieved from the protein data bank (PDB ID: 6VXX) (Figure 5). The residue alanine at position 930 though not associated with the active site of the molecule stabilizes majority of the chain A moiety in the protein due to its hydrophobic nature. On substitution with Valine in the same position, it can potentially change the affinity of the molecule toward its receptor.

Thus, taken together the mutations in S protein of Indian isolates can potentially alter virus entry and thus determine the infectivity of the virus. Also, S protein mutations that alter the surface epitopes, might lead to escape from recognition of host’s immune system/antibodies thereby challenging vaccine development if such sequences are included in the vaccine formulations. More sequence information along with mutational studies on receptor virus binding will further help strengthen this observation. The sites of mutation, geographic location, frequency of such mutations, knowledge about progression of infection, and disease severities will help correlating the significance of these mutation with respect to virus evolution and virulence. Such information will further help in identification and strategic designing of rational drug targets.

## Abbreviations

CFSSP: Chou and Fasman secondary structure prediction
CoV: coronavirus
RBD: receptor-binding domain
WHO: World Health Organization
